# Evidence that promotion of male circumcision did not lead to sexual risk compensation in prioritized Sub-Saharan countries

**DOI:** 10.1371/journal.pone.0175928

**Published:** 2017-04-25

**Authors:** Chyun-Fung Shi, Michael Li, Jonathan Dushoff

**Affiliations:** 1 Department of Biology, McMaster University, Hamilton, Ontario, Canada; 2 Department of Mathematics and Statistics, McMaster University, Hamilton, Ontario, Canada; 3 Institute for Infectious Disease Research, McMaster University, Hamilton, Ontario, Canada; International AIDS Vaccine Initiative, UNITED STATES

## Abstract

**Background:**

WHO and UNAIDS prioritized 14 eastern and southern African countries with high HIV and low male circumcision prevalence for a voluntary medical male circumcision (VMMC) scale-up in 2007. Because circumcision provides only partial protection against HIV infection to men, the issue of possible risk compensation in response to VMMC campaigns is of particular concern. In this study, we looked at population-level survey data from the countries prioritized by WHO for a VMMC scale-up. We compared the difference in sexual risk behaviours (SRB) between circumcised and uncircumcised men before and after the WHO’s official VMMC promotion.

**Materials and methods:**

Ten countries (Kenya, Lesotho, Malawi, Mozambique, Namibia, Rwanda, Tanzania, Uganda, Zambia and Zimbabwe) participating in the WHO’s VMMC scale-up had available data from the Demographic and Health Surveys (DHS). We used cumulative-link mixed models to investigate *interactions* between survey period and circumcision status in predicting SRB, in order to evaluate whether the *difference* between the behavior of the two groups changed before and after the scale-up, while controlling for socio-demographic and knowledge-related covariates. The main responses were condom use at last sex and number of non-cohabiting sexual partners, both in the last 12 months.

**Results:**

There was little change in condom use by circumcised men *relative to uncircumcised men* from before the VMMC scale up to after the scale up. The relative odds ratio is 1.06 (95% CI, 0.95–1.18; interaction P = 0.310). Similarly, there was little change in the number of non-cohabiting partners in circumcised men (relative to uncircumcised men): the relative odds ratio of increasing the number of partners is 0.95 (95% CI, 0.86–1.05; interaction P = 0.319). Age, religion, education, job, marital status, media use and HIV knowledge also showed statistically significant association with the studied risk behaviours. We also found significant differences among countries, while controlling for covariates.

**Conclusions:**

Overall, we find no evidence of sexual risk compensation in response to VMMC campaigns in countries prioritized by WHO. Changes in relative partner behaviour and the relative odds of condom use were small (and of uncertain sign). In fact, our estimates, though not significant, both suggest slightly less risky behavior. We conclude that sexual risk compensation in response to VMMC campaigns has not been a serious problem to date, but urge continued attention to local context, and to promulgating accurate messages about circumcision within and beyond the VMMC context.

## Background

After years of research into connections between male circumcision and HIV risk reduction [1–3], and three randomized clinical trials (RCT) which found that voluntary medical male circumcision (VMMC) interventions reduce men’s heterosexual acquisition of HIV by up to 60% [4–6], the World Health Organization (WHO) and the Joint United Nation Programme on HIV/AIDS (UNAIDS) recommended a VMMC scale-up as part of biomedical HIV prevention interventions in 2007 [7]. WHO subsequently prioritized 14 eastern and southern African countries with generalized heterosexual HIV epidemics and low circumcision prevalence for a VMMC scale-up, with a goal of 80% coverage of males aged 15-49 by 2015 [8]. By 2015, Kenya, Tanzania and Ethiopia (Gambella province) had surpassed the goal set in 2011, while other nations ranged from 10% to 70% coverage [9]. One concern with VMMC campaigns is sexual risk compensation [10], a danger exacerbated by the fact that in many places medical resources were insufficient to meet VMMC demand, likely leading people to seek services at sites where medical guidelines and safe-sex counseling were limited [11].

Sexual risk compensation could undercut the effectiveness of VMMC as an anti-HIV measure (particularly since circumcision is only partially protective [12]), and could also increase transmission risk of other sexually transmitted infections. Risk compensation is a cognitive-behavioral balance between risks (e.g., disease) and potential benefits (e.g., increased pleasure) of risky behaviours like unprotected sex [13–15]. Because circumcision is only partially protective for men from acquiring HIV (and has not been shown to directly protect females) [16], the issue of possible sexual risk compensation in response to VMMC is of particular concern [10, 12, 17–21]. While the RCTs and prospective studies found no significant changes in sexual risk behaviour (SRB) after VMMC efforts [4–6, 22–24], the cross-sectional studies in Uganda [25, 26] found positive associations.

For example, a study of 2011 Uganda AIDS Indicator Survey found that circumcised men were more likely to have more non-marital sex and more life-time sexual partners and less likely to use condom with a non-marital partner compared to uncircumcised men [25]. Another Ugandan study comparing changes of SRB from 2004 to 2011 using DHS data found statistically significant evidence for lower condom use among those circumcised in 2011 than in 2004 [26]. Qualitative studies found results ranging from no SRB in men with VMMC [27] to a minority of participants reporting increases in risk behavior in voluntarily medically circumcised men [28] and traditionally circumcised men [20].

In this study, we look at available population-level survey data from ten of the fourteen countries prioritized by the WHO for the African VMMC scale-up. Survey data provides a useful complement to RCT data, because it is not subject to artifacts that may occur in RCTs due to intense HIV counseling and education [29]. We believe this is the first population-scale investigation of whether the *difference* between the behavior of circumcised and uncircumcised men change from before to after the WHO’s VMMC scale-up.

## Materials and methods

This study focused on changes in sexual risk behavior (SRB) possibly associated with male circumcision, using one survey before and one survey after the 2007 VMMC recommendation from each country (see Table A and Table B in S1 Tables).

### Data collection and measurement

Nationally representative surveys from the Demographic and Health Surveys (DHS) were analyzed in this study. From the 14 countries prioritized for VMMC by WHO, we used the 10 that had SRB data from DHS in both time periods: Kenya, Lesotho, Malawi, Mozambique, Namibia, Rwanda, Tanzania, Uganda, Zambia and Zimbabwe. Males older than 49 years old were excluded because they were not covered by WHO guidelines [8] as well as those who reported never having had sex. Lesotho was excluded from the condom analysis because condom data was not available in the pre-2008 survey. We did not attempt to correct for survey sampling weights in doing inferential analysis; thus, our inferences formally apply to the surveyed population rather than the national populations.

For our main response variables, we selected SRB variables used in related studies (e.g., [4–6, 22]) that were available from DHS: condom use at last sex and number of reported non-cohabiting sexual partners in the last 12 months (that is, we excluded partners that respondents were married to or living with).

Our main predictor was male circumcision status: circumcised or uncircumcised. Additional covariates were age, education, work status, religion, wealth, residence, marital status, media use, HIV knowledge, survey period (i.e., pre-2008 vs. post 2008) and country (see Table A and Table B in S1 Tables for details). We included media use because media consumption is known to correlate with health behavior (e.g., [30, 31]), and there were concerns about how the media provided information about VMMC in sub-Saharan Africa, and resulting perceptions [32, 33]. HIV knowledge was included because inaccurate knowledge was shown to correlate with SRB (e.g., [10, 34, 35])

We decided *a priori* to code the DHS raw wealth index using a three-knot spline, and individual age using a four-knot spline. Marital status was recoded into five categories, and religion into four categories. We constructed overall indices for media use and HIV knowledge by using the first component from a scaled, uncentered principal component analysis (PCA); we did not further analyze the PCA results. (For coding and summary information, see Table A, Table B in S1 Tables).

#### Statistical model

We compared how circumcised men changed through time *relative* to how uncircumcised men changed through time. This is equivalent to asking how the *difference* between the groups changed from the pre- to post-VMMC survey. We did this using interaction terms from the fitted models [36]). We exponentiated the interaction terms from our cumulative link models so that we could interpret them as relative odds ratios (i.e., ratios between odds ratios). The ordinal package in R was used to fit binary (condom use) and ordinal (numbers of non-cohabiting sexual partners) responses to our predictors and covariates, while using cluster as a random effect, to control for correlations between individuals from the same geographic area and background. A random effect for the response to media use was added to the models, because we believed that media content in each country was likely to be different [33].

Instead of calculating several P values for each variable (using contrasts or a baseline level), we calculated variable-level P values using the anova function in R. “Prediction” plots were made by calculating the effect of each level or value of a predictor variable on the linear predictor of the model, using the model center as a reference point. Any sample with missing data for a given variable was adaptively dropped from analyses involving that variable.

#### Scripts

All of the R scripts used to analyze the data and produce the figures will be made available on the web when the paper is published. Data is available on request from dhsprogram.com.

## Results

We analyzed responses from 67,590 men (24,974 before 2008 and 42,616 after 2008). Overall, circumcision prevalence was 33% in the pre-2008 surveys (Table A in S1 Tables) and 43% in the after-2008 surveys (Table B in S1 Tables). Kenya had the highest prevalence (88% and 94%) and Zimbabwe the lowest (12% and 11%) among the surveyed nations. Circumcision prevalence decreased from pre-2008 to post-2008 in Malawi, Mozambique, and Zimbabwe. For detailed sample characteristics, see Table A and Table B in S1 Tables.

**Fig 1:**
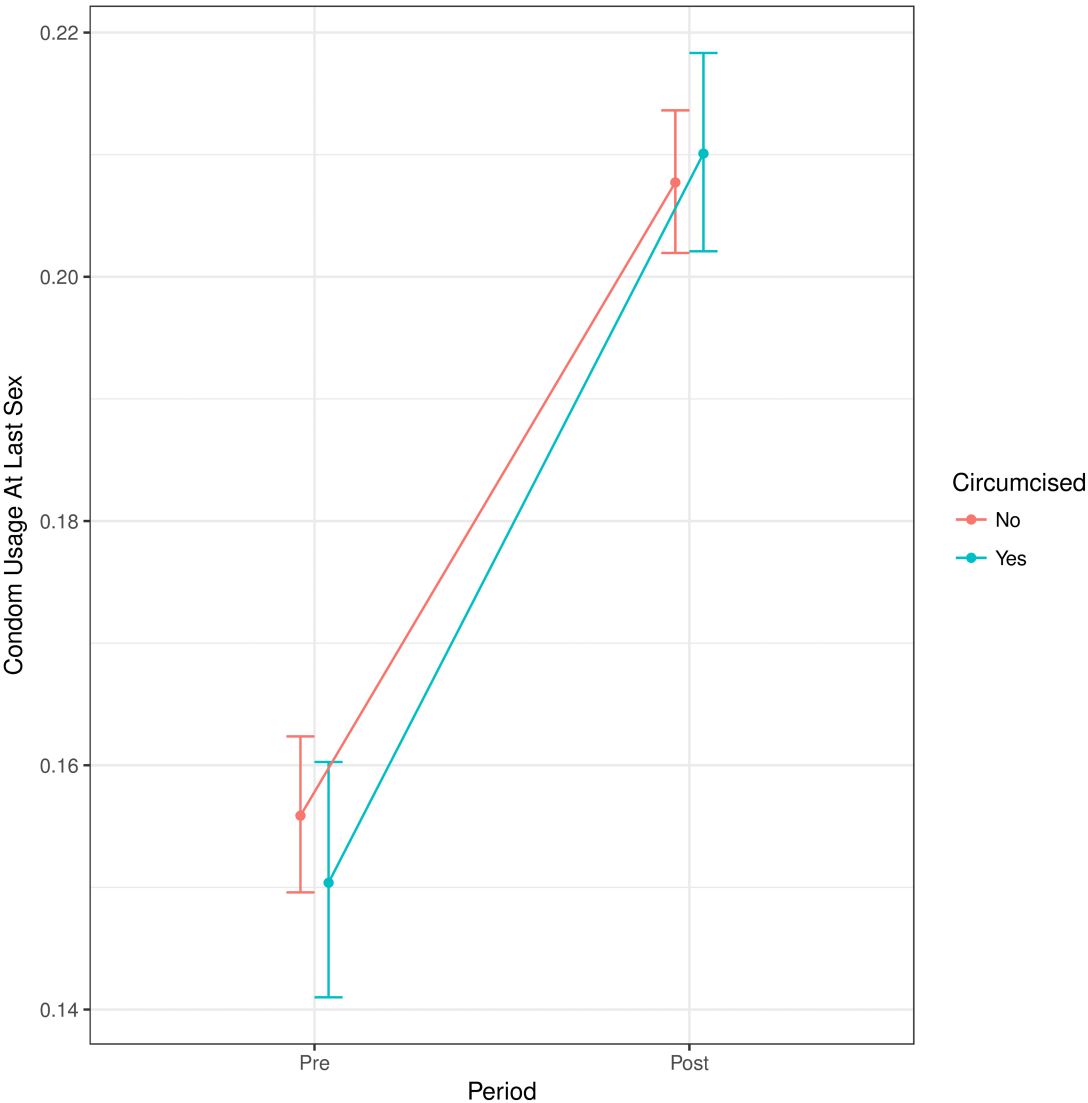
The effect of circumcision status on condom use at last sex before and after the WHO’s VMMC scale-up campaign. The *relative* odds ratio is higher by a factor of 1.06 than the corresponding odds ratio (95% CI, 0.95–1.18; interaction P = 0.310). Groups are offset horizontally for readability.

Fig 1 shows our analysis of condom use. Condom use by circumcised men *relative to uncircumcised men* increased between surveys but the difference was not significant. This was shown by the reduced gap (and change of direction) between the groups in the later period. The relative odds ratio is 1.06 (95% CI, 0.95–1.18; interaction P = 0.310)– this means that the odds ratio of a circumcised man (compared to an uncircumcised man) using a condom in the later period is 1.06 times higher than in the earlier period. The figure also shows an overall increase in condom use through time.

**Fig 2:**
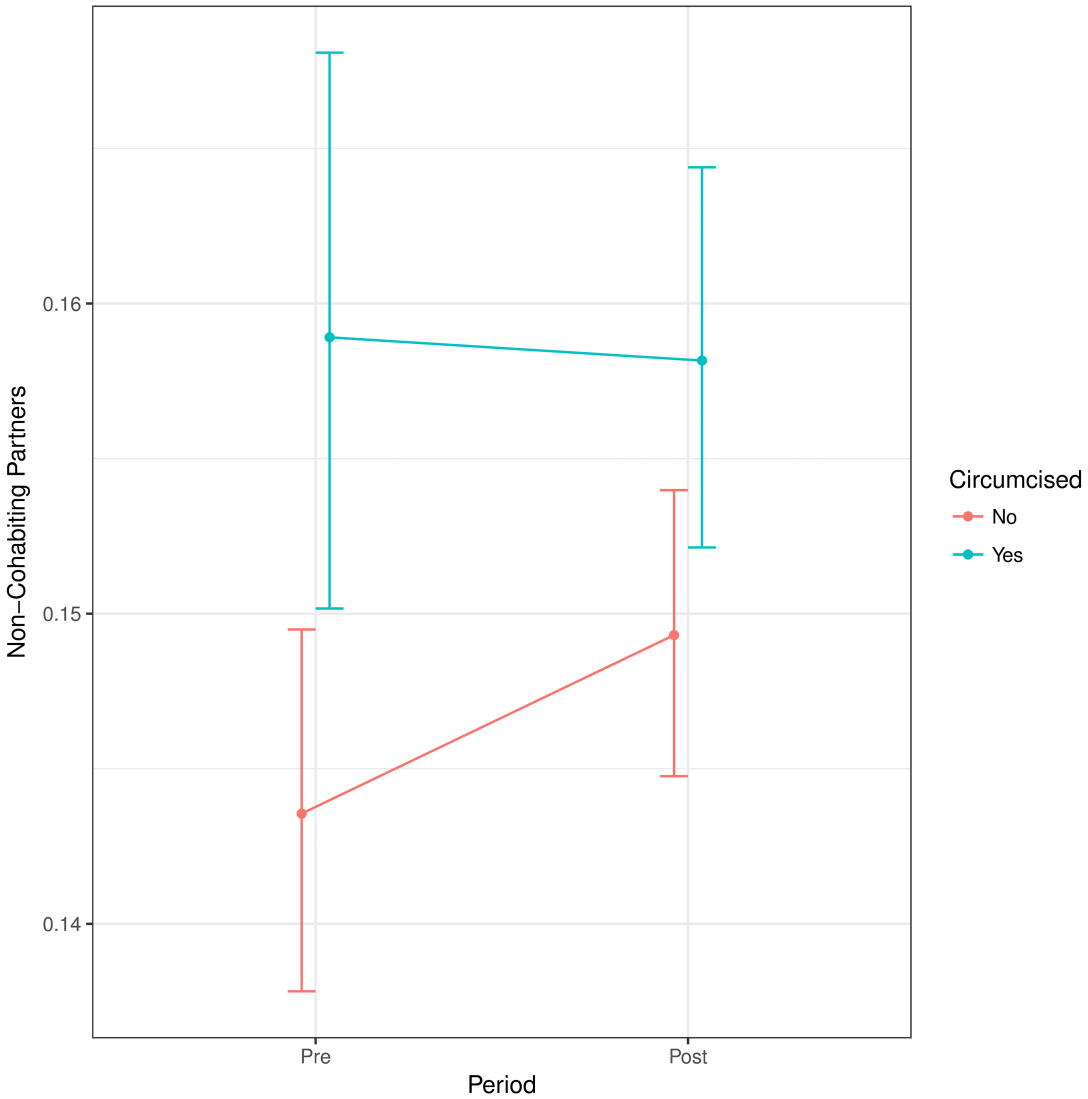
The effect of circumcision status on number of non-cohabiting partners before and after the WHO’s VMMC scale-up campaign. The *relative* odds ratio of a circumcised man taking an extra sexual partner in the later period is 0.95 (95% CI, 0.86–1.05; interaction P = 0.319).

Fig 2 shows the effect of circumcision status on the number of non-cohabiting sexual partners within the previous year. We find little change in the behaviour of circumcised men (relative to uncircumcised men) through time: the relative odds ratio of increasing the number of partners is 0.95 (95% CI, 0.86–1.05; interaction P = 0.319). In other words, the odds ratio between circumcised and uncircumcised men is similar (on average) in the earlier and the later surveys.

**Fig 3:**
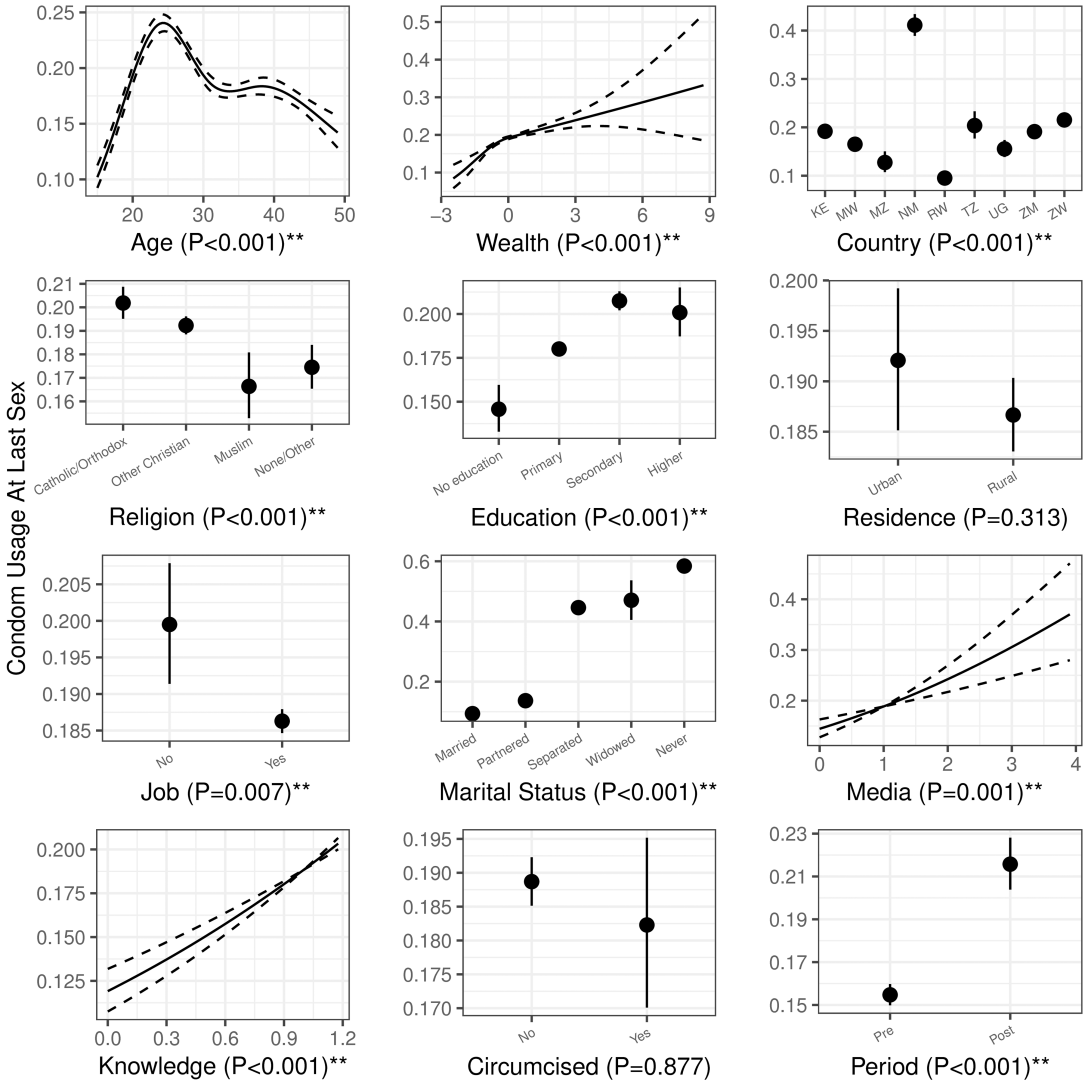
The effect of covariates on condom use at last sex. Solid lines and dash lines show the effect and 95% prediction interval for continuous covariates. Solid points and vertical solid lines show the effect and 95% prediction interval for discrete covariates. Variable-level P values are included for each covariate.

**Fig 4:**
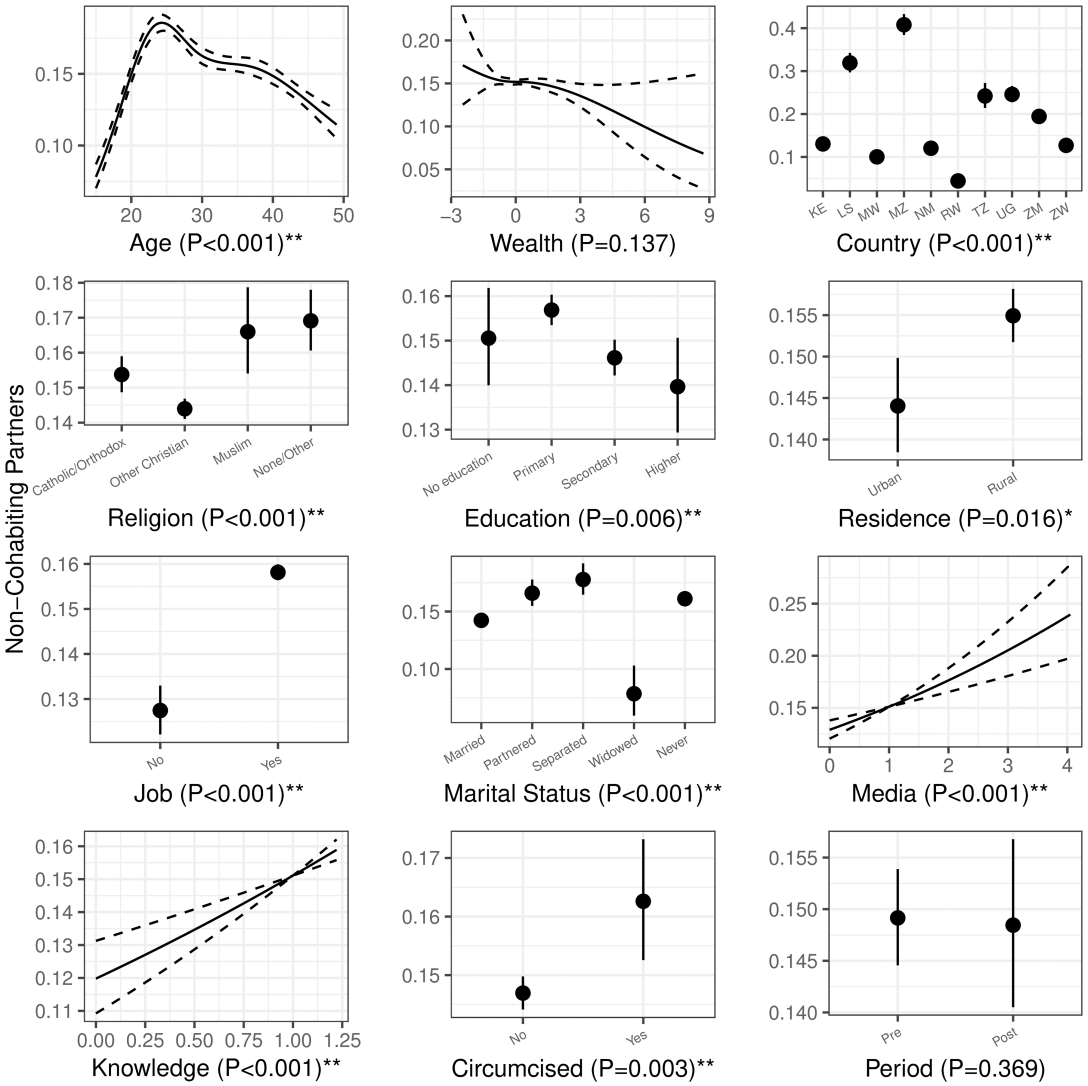
The effect of covariates on reported non-cohabiting partners in the previous year. See Fig 3 for details.

Patterns of how risk behaviors were affected by the covariates, with variable-level P-values, are shown in Figs 3 and 4. These patterns are from the multivariate mixed-model analysis: they control for primary predictor, other covariates, and the random effects of survey cluster.

We found a statistically significant effect of country in predicting sexual risk behaviors (P<0.001 for both behaviors). Both of our information-related factors (media use and HIV knowledge) were positively correlated with more condom use (decreased risk), but also with more sexual partners (increased risk). Most of the socio-demographic factors, (i.e., age, religion, education, job, and marital status) were significantly associated with both risk behaviours. Wealth was significantly associated only with condom use, and residence only with number of partners. Both condom use and number of partners peak when age is around the mid-20s. Married and partnered people report low rates of condom use, but (perhaps surprisingly) not low numbers of non-cohabiting partners.

## Discussion

Although many researchers have expressed concern that VMMC interventions may lead to increased risk compensation, our analysis of 10 nations (Kenya, Lesotho, Malawi, Mozambique, Namibia, Rwanda, Tanzania, Uganda, Zambia and Zimbabwe) participating in the WHO’s VMMC scale-up campaign [9] found no evidence of increased risky behavior by circumcised men (relative to uncircumcised) men when comparing post- and pre-intervention surveys. Our estimates indicate that the relative amount of risky behaviour *decreased*, and confidence intervals show that if there was an increase we did not observe, it must have been small (See Figs 1 and 2). These results are consistent with posttrial studies from VMMC RCTs in Kenya [23, 24] and Uganda [22, 37].

We saw an overall increase in condom use from pre- to post-intervention (See Figs 1 and 3). This could be due to any number of factors, including improved knowledge or fear of HIV; improved health education; or improved access to condoms. We do not interpret this increase as being informative about VMMC campaigns. The increase in condom use is consistent with longitudinal results from Nyanza, Kenya [24] and the cross-sectional analysis in Kisumu, Kenya [38], but a post-trial study in Uganda found a significant decrease of condom use in both male circumcision acceptors and nonacceptors [37]. Importantly, many factors are expected to affect our long-term, large-scale comparisons. Unlike changes in the *difference* between circumcised and uncircumcised men, our study does not postulate that *overall* changes provide information about the effects of the VMMC campaign.

Overall, circumcised men had more non-cohabiting partners than uncircumcised men (the Uganda post-trial followup did not find a significant difference in this regard [37]). However, the difference between the groups changed very little from pre- to post-intervention surveys, providing evidence against sexual risk compensation. while the overall difference between before and after the VMMC scale-up was not obvious (see Fig 4).

We also found differences in sexual risk behaviors between countries, after controlling for available covariates). For example, Mozambique showed high sexual risk behaviors (low condom use and the highest number of non-cohabiting sexual partners, see Figs 3 and 4). More broadly, circumcision culture and values varied in different societies [39, 40]; these factors could contribute to different sexual risk behaviors in different countries.

We found several suggestive effects of socio-demographic factors (see Figs 3 and 4). First, more time spent on media was associated with both more condom usage, and more sexual partners. Being in the mid-20s and better HIV knowledge had similar effects. It’s possible that these groups are using condoms more in whole or in part because they are aware of the risk associated with additional sexual partners. Second, men with limited education tended to perform riskier sexual behaviors (i.e., more sexual partners and less likely to use condom). Similar results were found in men from rural areas and men who reported being employed. Third, men cohabiting with partners without formal marriage were among the most risky groups, having more sexual partners and less prone to use condom. Understanding which groups are at highest risk can be useful in order to deliver public-health messages effectively to tailored targets [40].

### Limitations

Since the study is based on observation, not experiment, we are unable to make direct conclusions about causality; nor could we draw a comparison of male circumcised for religious reason versus those circumcised for protection against HIV infection. Our analysis was based on DHS data which did not inquire about samples’ sexual orientation. In general, the role of homosexuality in Africa is understudied, and difficult to study; more such research is called for [41]. Our study focuses on a simple comparison between two time periods in each country; conclusions about VMMC are purely correlational. We attempted to mitigate this concern by focusing only on *differences* between circumcised and uncircumcised men, while controlling for important covariates.

## Conclusion

We looked for evidence of sexual risk compensation across prioritized African countries, by comparing relative risk behaviour between circumcised and uncircumcised men before and after the WHO VMMC scale-up. We found evidence that VMMC campaigns are associated with little or no sexual risk compensation in the variables we measured. This is an encouraging sign. Nonetheless, several caveats should be mentioned. First, because HIV knowledge was shown to correlate with SRB, it is important to affirm accurate media coverage that circumcision provides only partial protection against HIV infection and that safe sex behavior is still warranted [32, 33]. Second, knowledge about benefits and limitations of circumcision should be transferred to reach those who were circumcised outside the VMMC setting, either, for example by traditional healers [18, 42]. Last but not least, VMMC initiatives should remain aware of local context. Our results showing substantial differences based on country and religion reinforce this point.

## Supporting information

S1 Tables Sample characteristics. (PDF)
