# Supplementary material for: Evidence that promotion of male circumcision did not lead to sexual risk compensation in prioritized Sub-Saharan countries
Source: PLoS One. 2017 Apr 25;12(4):e0175928. doi: 10.1371/journal.pone.0175928 (PMC5404849; doi:10.1371/journal.pone.0175928)
Supplement: S1 Tables — (PDF) [file pone.0175928.s001.pdf]

S1 Tables: Sample Characteristics

Table A. Sample Characteristics of pre-2008 Surveys.

Country	KE	LS	MW	MZ	NM	RW	TZ	UG	ZM	ZW	Total
**Survey Year**	2003	2004	2004	2003	2006-7	2005	2004-5	2006	2007	2005-6	
**Sample Size**	2210	1845	2311	1391	2684	2346	1859	1694	4380	4254	24974
**Category (in percentage)**											
**Age Group**											
15-19	9.6	17.7	9.6	19.6	12.1	2.3	10.1	8.0	9.9	8.1	10.1
20-24	19.3	22.1	18.5	20.1	19.1	11.9	17.0	15.7	16.3	19.6	17.9
25-29	18.0	17.6	23.1	16.1	20.2	18.8	17.8	17.9	20.1	20.2	19.4
30-34	16.9	14.9	18.8	13.2	17.3	18.7	18.0	20.2	19.8	19.5	18.2
35-39	15.6	11.3	11.3	12.8	13.4	17.3	14.5	17.1	15.5	13.9	14.4
40-44	12.7	8.4	11.7	10.0	10.9	16.3	13.8	12.6	10.0	9.7	11.4
45-49	7.9	8.1	7.0	8.3	7.0	14.7	8.8	8.4	8.4	9.1	8.8
**Residence**											
Urban	35.6	27.3	15.8	52.6	45.9	22.4	21.9	15.6	40.2	35.6	32.4
Rural	64.4	72.7	84.2	47.4	54.1	77.6	78.1	84.4	59.8	64.4	67.6
**Education**											
No education	4.8	17.1	12.0	7.2	10.4	21.1	13.1	6.2	5.2	1.6	8.9
Primary	52.7	53.0	63.0	66.0	28.1	64.2	74.3	65.5	47.2	28.7	50.3
Secondary	28.8	26.7	22.9	25.0	54.1	12.5	9.3	21.0	39.4	62.6	34.7
Higher	13.7	3.2	2.1	1.8	7.5	2.1	3.4	7.3	8.2	7.1	6.1
**Religion**											
Catholic/Orthodox	28.0	44.4	21.4	37.4	28.9	48.0	25.3	43.2	20.2	10.7	27.6
Other Christian	58.1	34.5	23.8	28.7	68.3	47.1	22.4	43.2	76.4	47.6	49.4
Muslim	7.6	0.0	14.8	33.7	0.0	2.3	41.2	11.5	0.4	0.9	8.2
None/Other	6.3	21.1	39.9	0.2	2.7	2.6	11.1	2.2	3.0	40.9	14.8
**Marital Status**											
Never Unioned	27.8	45.0	18.2	33.2	52.9	10.4	22.5	17.5	25.0	24.5	27.4
Married	67.3	47.8	77.6	25.9	23.7	56.0	66.2	69.4	70.2	66.5	59.2
Partnered	1.1	0.4	1.3	36.8	18.6	31.3	5.9	7.9	0.6	3.8	9.0
Separated	3.2	4.7	2.6	4.0	4.4	1.9	4.7	4.9	3.6	4.3	3.8
Widowed	0.5	2.1	0.2	0.1	0.4	0.4	0.6	0.4	0.7	0.9	0.7
**Job**											
No	14.4	63.4	33.7	36.4	28.6	41.9	7.1	1.9	13.1	23.9	25.1
Yes	85.6	36.6	66.3	63.6	71.4	58.1	92.9	98.1	86.9	76.1	74.9
**Condom Usage at Last Sex**											
No	81.4	NA	84.7	78.6	42.7	93.1	81.3	82.2	76.1	74.4	70.4
Yes	18.6	NA	15.3	21.4	57.3	6.9	18.7	17.8	23.9	25.6	22.2
**Non-cohabiting Partners**											
0	83.9	73.2	88.0	59.6	84.5	95	71.1	70.7	79.4	86.5	81.0
1	13.1	20.2	10.7	27.9	12.8	5	22.8	23.9	17.3	11.4	15.3
>1	3.0	6.7	1.3	12.5	2.6	0	6.2	5.4	3.3	2.0	3.6
**Circumcised**											
No	12.4	44.1	73.5	45.1	77	87.8	24.2	74.6	82.7	88.3	66.6
Yes	87.6	55.9	26.5	54.9	23	12.2	75.8	25.4	17.3	11.7	33.4
**HIV Knowledge**											
**Condoms Protect**											
No	12.1	15.1	19.6	6.3	10.2	6.3	16.2	8.4	20.6	15.2	14.0
Yes	81.9	74.6	77.7	90.0	88.4	90.8	78.6	86.4	77.2	83.5	82.5
Don't know	6.0	10.3	2.6	3.7	1.3	2.9	5.2	5.2	2.2	1.3	3.5
**Less Partner Protect**											
No	2.5	9.6	12.2	9.1	5.3	9.2	9.7	3.3	9.1	11.9	8.6
Yes	97.0	83.5	86.0	84.9	93.0	89.4	88.4	95.7	90.4	87.5	89.7
Don't know	0.5	6.9	1.8	6.0	1.8	1.4	1.8	0.9	0.5	0.6	1.8
**Healthy People Get Aids**											
No	3.6	16.9	5.4	8.3	4.7	3.0	8.0	6.7	10.2	5.6	7.1
Yes	95.4	75.0	93.7	88.6	93.3	95.4	87.8	91.0	88.6	93.8	90.8
Don't know	1.0	8.1	0.9	3.0	2.0	1.5	4.1	2.3	1.2	0.6	2.1
**Media**											
**Newspaper and Magazines**											
Not at all	24.5	72.9	49.3	57.4	29.2	65.1	40.8	60.7	53.1	37.6	47.4
Less than once a week	24.5	9.8	25.6	23.7	20.5	22.0	25.6	19.0	18.1	22.5	21.1
At least once a week	29.3	11.8	15.1	5.2	29.7	10.9	23.3	14.1	17.7	24.9	19.4
Almost every day	21.7	5.5	10.0	13.7	20.6	2.0	10.3	6.1	11.1	14.9	12.1
**Radio**											
Not at all	3.0	37.2	4.9	3.5	6.9	7.8	7.0	6.7	13.3	23.2	12.4
Less than once a week	3.5	7.5	9.3	16.6	7.7	12.7	10.2	5.3	11.7	13.0	10.0
At least once a week	8.7	15.0	13.7	1.7	15.6	24.1	20.7	21.7	19.0	17.6	16.5
Almost every day	84.8	40.3	72.2	78.3	69.8	55.5	62.2	66.4	56.0	46.2	61.1
**TV**											
Not at all	32.8	73.3	66.4	49.5	38.3	75.3	50.1	71.8	53.5	47.7	54.5
Less than once a week	22.5	6.3	16.8	16.2	12.0	13.8	24.5	14.2	12.9	11.5	14.5
At least once a week	17.9	13.2	9.0	3.0	14.2	6.9	15.0	8.4	10.3	13.1	11.5
Almost every day	26.8	7.1	7.8	31.3	35.5	4.0	10.5	5.6	23.3	27.7	19.5

The following samples were excluded from this study: never heard of AIDS, never had sexual intercourse prior to the surveys, unaware of their circumcision status, or had NA answers to any of the variables. Media use referred to frequency samples reported on time spending on newspaper, radio and television. HIV knowledge referred to questions of "Reduce risk of getting HIV: always use condoms during sex," "Reduce chance of AIDS: have one sex partner with no other partner," and "A healthy looking person can have HIV."

Table B. Sample Characteristics of Post-2008 Surveys

Country	KE	LS	MW	MZ	NM	RW	TZ	UG	ZM	ZW	Total
**Survey Year**	2014	2014	2010	2011	2013	2014-15	2010	2011	2013-14	2011	
**Sample Size**	8650	1975	4905	3109	2844	3166	1729	1567	9993	4678	42616
**Category (in percentage)**											
**Age Group**											
15-19	8.1	16.4	11.3	17.3	9.8	2.4	9.7	9.3	11.4	7.2	10.0
20-24	15.7	21.2	16.2	19.8	20.9	12.6	15.0	14.7	15.4	17.7	16.5
25-29	19.5	18.1	19.4	17.6	18.5	24.6	16.3	20.9	17.2	21.3	19.2
30-34	18.3	15.5	18.0	14.9	15.8	21.0	17.4	18.5	17.8	18.8	17.8
35-39	15.9	11.9	15.0	13.5	13.6	14.5	16.6	16.8	15.7	16.2	15.2
40-44	12.9	10.0	10.6	8.8	12.2	13.0	13.7	11.0	13.1	11.3	12.0
45-49	9.6	6.9	9.5	7.9	9.2	11.9	11.3	8.9	9.3	7.5	9.2
**Residence**											
Urban	40.4	32.4	13.2	45	52.3	19.5	23.4	28.9	44.1	33.9	35.5
Rural	59.6	67.6	86.8	55	47.7	80.5	76.6	71.1	55.9	66.1	64.5
**Education**											
No education	6.1	9.6	7.0	12.4	10.0	14.7	11.0	5.8	4.2	0.9	6.9
Primary	51.4	46.1	62.8	52.4	22.8	69.0	68.4	56.5	41.6	24.0	47.5
Secondary	29.4	35.7	27.2	31.2	58.4	13.9	19.3	27.1	45.3	67.3	37.7
Higher	13.1	8.6	3.0	4.1	8.8	2.5	1.3	10.6	8.8	7.8	7.8
**Religion**											
Catholic/Orthodox	22.8	40.1	20.8	28.6	26.3	47.0	0	45.4	19.5	9.8	23.5
Other Christian	62.5	46.2	25.7	32.2	57.2	48.5	0	39.9	78.6	50.5	53.0
Muslim	10.0	0.4	10.9	17.9	0.0	1.8	0	13.2	0.7	0.7	5.5
None/Other	4.6	13.3	42.6	21.4	16.5	2.6	0	1.4	1.2	39.0	13.9
Tanzanian	0.0	0.0	0.0	0.0	0.0	0.0	100	0.0	0.0	0.0	4.1
**Marital Status**											
Never Unioned	26.6	45.7	20.3	29.5	55.5	13.8	22.5	19.6	26.9	23.2	27.2
Married	65.1	46.5	65.3	42.1	21.7	60.4	66.7	54.8	68.7	70.6	60.5
Partnered	2.7	1.1	11.5	22.3	19.5	23.6	4.0	20.6	0.8	1.2	7.8
Separated	5.2	5.1	2.7	5.4	3.1	2.0	5.8	4.7	3.5	4.1	4.0
Widowed	0.3	1.6	0.2	0.7	0.2	0.2	0.9	0.3	0.2	0.8	0.4
**Job**											
No	9	37.4	11.8	13.9	33.7	2.2	5	4.4	15.9	29.3	15.6
Yes	91	62.6	88.2	86.1	66.3	97.8	95	95.6	84.1	70.7	84.4
**Condom Usage at Last Sex**											
No	70.8	40.8	78.9	74.8	41.3	84.6	79.7	79.1	73.6	71.8	71.1
Yes	29.2	59.2	21.1	25.2	58.7	15.4	20.3	20.9	26.4	28.2	28.9
**Non-cohabiting Partners**											
0	84.0	66.5	86.9	61.9	86.5	92.9	72.8	73.8	79.3	84.8	80.9
1	13.6	26.8	11.7	30.8	11.8	6.3	23.0	22.6	18.1	13.1	16.3
>1	2.4	6.7	1.4	7.3	1.7	0.9	4.2	3.6	2.6	2.1	2.8
**Circumcised**											
No	6.4	21	79.3	50.3	71.3	84.8	20.4	70.8	75.2	89.4	57
Yes	93.6	79	20.7	49.7	28.7	15.2	79.6	29.2	24.8	10.6	43
**HIV Knowledge**											
**Condoms Protect**											
No	10.1	8.7	25.0	17.0	7.3	4.6	19.7	13.8	11.8	12.7	12.9
Yes	87.4	89.5	73.8	78.6	91.7	94.6	75.8	83.5	87.2	85.8	85.3
Don't know	2.4	1.9	1.1	4.4	1.1	0.8	4.5	2.7	1.0	1.5	1.8
**Less Partner Protect**											
No	4.8	8.6	12.1	6.5	4.4	16.7	7.9	6.5	3.2	5.3	6.7
Yes	94.3	88.7	87.5	88.4	94.1	81.6	90.5	92.6	96.3	93.8	92.1
Don't know	0.9	2.7	0.4	5.0	1.4	1.7	1.7	0.9	0.4	0.9	1.3
**Healthy People Get Aids**											
No	6.4	10.0	5.8	6.5	6.1	4.7	7.6	5.9	10.7	9.1	7.7
Yes	92.1	87.9	93.9	87.2	92.0	94.6	90.7	92.9	88.3	90.0	90.8
Don't know	1.5	2.1	0.3	6.3	1.9	0.7	1.7	1.2	1.0	0.9	1.5
**Media**											
**Newspaper and Magazines**											
Not at all	42.3	66.7	55.2	61.4	87.7	69.3	49.1	51.7	49.7	36.8	53.1
Less than once a week	21.6	17.3	21.3	19.2	7.7	22.0	20.1	19.0	19.1	31.4	20.6
At least once a week	36.1	16.1	17.5	19.5	4.5	8.7	22.7	20.4	18.5	31.8	22.0
Almost every day	0.0	0.0	6.0	0.0	0.0	0.0	8.1	8.9	12.7	0.0	4.3
**Radio**											
Not at all	6.9	27.1	9.7	11.0	13.2	2.8	12.9	6.8	18.9	26.6	13.8
Less than once a week	9.0	16.9	12.0	22.6	25.1	10.1	8.0	6.3	14.5	24.3	14.7
At least once a week	84.1	56.1	19.6	66.4	61.7	87.0	17.1	15.4	21.6	49.1	49.1
Almost every day	0.0	0.0	58.7	0.0	0.0	0.0	62.1	71.5	45.0	0.0	22.4
**TV**											
Not at all	26.3	54.8	45.7	38.5	38.9	42.8	41.1	51.6	45.5	36.2	39.9
Less than once a week	18.8	18.1	24.0	23.0	17.2	32.5	19.7	15.7	15.3	23.4	20.2
At least once a week	54.9	27.1	18.8	38.5	44.0	24.7	20.4	12.4	11.8	40.4	30.6
Almost every day	0.0	0.0	11.5	0.0	0.0	0.0	18.8	20.3	27.5	0.0	9.3

The 2010 Tanzanian survey omitted data about religion; we used a dummy religion (Tanzanian), so that this survey could be included. See S1 Table A for reference.
